# Supplementary material for: Complexome profiling of the Chlamydomonas psb28 mutant reveals TEF5 as an early PSII assembly factor
Source: Plant Cell. 2025 Mar 17;37(6):koaf055. doi: 10.1093/plcell/koaf055 (PMC12164590; doi:10.1093/plcell/koaf055)
Supplement: koaf055_Supplementary_Data [file koaf055_supplementary_data.zip › psb28_interactive.zip!/plots/intensity/qualityPlots/peptides/CPN60C_Cre06.g309100.t1.2.html]

### Cre06.g309100.t1.2

Trivial name: CPN60C  
SeqCoverage [median]: 72.50  
Mass: 60.47 kDa  
  
 

| Sequence | MissedCleavage | Mass [Da] | wtIntensity | muIntensity | Score |
| --- | --- | --- | --- | --- | --- |
| AAMDATTSDYDR | 0 | 1315.54 | 1230.38 | 2807.46 | 157.24 |
| AAMDATTSDYDREK | 1 | 1572.67 | 35316.10 | 41632.21 | 167.01 |
| AAMDATTSDYDREKLQER | 2 | 2098.96 | 1293.89 | 13557.70 | 61.73 |
| AAVEEGIVPGGGSALLHASK | 0 | 1861.99 | 91871.06 | 316562.98 | 151.83 |
| AIELKDKFENIGASLVK | 2 | 1874.05 | 23230.16 | 106819.07 | 140.81 |
| AKMISTTEEIAQVGTISANGER | 1 | 2305.16 | 0.00 | 13899.31 | 90.19 |
| APGFGDNR | 0 | 832.38 | 8749.16 | 25427.79 | 117.18 |
| APGFGDNRK | 1 | 960.48 | 0.00 | 2569.24 | 75.11 |
| DDTIVLHGAGAK | 0 | 1195.62 | 2706.16 | 11563.84 | 134.25 |
| DKFENIGASLVK | 1 | 1319.71 | 21475.90 | 64193.82 | 210.98 |
| DKVLAGVNK | 1 | 942.55 | 841.52 | 9452.25 | 118.50 |
| DRVVDALNATK | 1 | 1200.65 | 886.73 | 6277.88 | 95.09 |
| EGVITVNDGK | 0 | 1030.53 | 20290.12 | 49496.83 | 172.23 |
| EGVITVNDGKTLENELEVVEGMKFDR | 2 | 2920.45 | 2863.28 | 8845.56 | 65.07 |
| EIGELIAR | 0 | 899.51 | 56626.97 | 107324.13 | 176.66 |
| GGIIDPLK | 0 | 811.48 | 29518.05 | 76985.48 | 123.69 |
| GGIIDPLKVVR | 1 | 1165.72 | 8694.73 | 66917.22 | 102.52 |
| GINMAVEHVVGVLK | 0 | 1464.81 | 41022.52 | 214288.79 | 155.09 |
| GRNVMIEQTYGGPK | 1 | 1548.77 | 1058.26 | 4952.14 | 63.18 |
| GYISPYFVTDQK | 0 | 1416.69 | 24409.88 | 148506.00 | 215.72 |
| IGGASEVEVGEK | 0 | 1173.59 | 15122.11 | 37925.81 | 197.79 |
| IGGASEVEVGEKK | 1 | 1301.68 | 21824.44 | 38300.90 | 149.69 |
| IGGASEVEVGEKKDR | 2 | 1572.81 | 38796.73 | 81963.77 | 176.56 |
| IGVNIIQNALR | 0 | 1209.72 | 0.00 | 66332.53 | 203.02 |
| IGVNIIQNALRVPMK | 1 | 1664.98 | 986.47 | 21832.18 | 82.07 |
| ISGLQSLLPVLEK | 0 | 1395.83 | 10431.71 | 34573.59 | 226.04 |
| ITVTKDDTIVLHGAGAK | 1 | 1737.96 | 85938.90 | 165383.47 | 219.97 |
| KPAAGYPPAPDMY | 1 | 1376.64 | 3185.80 | 7958.72 | 151.22 |
| LADAVQVTLGPK | 0 | 1210.69 | 62649.40 | 190670.22 | 229.82 |
| LDNFDQK | 0 | 878.41 | 19921.79 | 54465.11 | 189.53 |
| MENVDVR | 0 | 861.40 | 3620.86 | 11417.23 | 127.02 |
| MISTTEEIAQVGTISANGER | 0 | 2106.03 | 633.99 | 3910.38 | 75.55 |
| NVMIEQTYGGPK | 0 | 1335.65 | 47899.73 | 88270.75 | 174.98 |
| QVASATNDVAGDGTTTATVLTR | 0 | 2148.07 | 38962.04 | 62753.80 | 180.63 |
| RGINMAVEHVVGVLK | 1 | 1620.91 | 2534.47 | 6265.30 | 106.17 |
| RISGLQSLLPVLEK | 1 | 1551.93 | 2170.67 | 0.00 | 73.72 |
| RITVTKDDTIVLHGAGAK | 2 | 1894.06 | 5869.53 | 25382.22 | 187.39 |
| SVAAGMNPMDLR | 0 | 1260.60 | 16215.31 | 29979.11 | 144.70 |
| SVAAGMNPMDLRR | 1 | 1416.70 | 3013.20 | 23953.58 | 88.19 |
| TIASNAGVEGAVIVGK | 0 | 1484.82 | 16918.50 | 52871.17 | 273.27 |
| TLDDVTAK | 0 | 861.44 | 36195.90 | 73327.48 | 254.37 |
| TLDDVTAKLDNFDQK | 1 | 1721.85 | 29064.40 | 91606.53 | 157.68 |
| TLENELEVVEGMK | 0 | 1489.73 | 543.23 | 0.00 | 85.36 |
| TLENELEVVEGMKFDR | 1 | 1907.93 | 24584.35 | 96331.48 | 117.98 |
| VGKEGVITVNDGK | 1 | 1314.71 | 12995.30 | 32301.72 | 132.86 |
| VLAGVNK | 0 | 699.43 | 11226.65 | 26282.05 | 138.61 |
| VLEMAEPQMGYNAATGEFVDMIK | 0 | 2543.17 | 10317.04 | 44760.63 | 104.67 |
| VPMKTIASNAGVEGAVIVGK | 1 | 1940.08 | 699.83 | 7649.88 | 93.50 |
| VVDALNATK | 0 | 929.52 | 95005.09 | 189171.81 | 138.54 |
|  |  |  | 989412.4 | 2857721.1 |  |
